# Pediatric hypertension as an early manifestation of cardiovascular disease in children

**DOI:** 10.1590/2175-8239-JBN-2023-0159en

**Published:** 2024-05-03

**Authors:** Vera Hermina Kalika Koch, Erika Arai Furusawa

**Affiliations:** 1 Universidade de São Paulo, Faculdade de Medicina, Departamento de Pediatria, São Paulo, SP, Brazil. Universidade de São Paulo Faculdade de Medicina Departamento de Pediatria São Paulo SP Brazil; 2 Universidade de São Paulo, Faculdade de Medicina, São Paulo, SP, Brazil. Universidade de São Paulo Faculdade de Medicina São Paulo SP Brazil

**Keywords:** Hypertension, Child, Cardiovascular Abnormalities

## Abstract

In adults, cardiovascular events associated with arterial hypertension (AH) have a major impact on morbidity and mortality. In light of recent findings, AH in children has been interpreted as early cardiovascular disease (CVD), while exposure to CV risk factors in children proves to be a predictor of subclinical CVD in adults. The American College of Cardiology/American Heart Association has recently updated the classifications for measuring blood pressure (BP) in adults and children. Primary AH in children is generally asymptomatic, and it is associated with a family history of AH, overweight/obesity, and normal morphofunctional characteristics of the urinary system. The younger the child and the higher the BP, the greater the likelihood of secondary AH. The investigation into the etiology of AH begins with a detailed anamnesis, which should include clinical information and details on the use of medication, smoking, and alcohol consumption from the perinatal period to the time of consultation. Modifying risk factors by reducing weight, decreasing alcohol consumption and increasing vegetable intake from childhood to adulthood has been associated with the resolution of AH in the childhood-adulthood transition, and with the reversal of cardiometabolic adverse effects in non-obese adult individuals. Pharmacological therapy should be initiated in cases of symptomatic AH, AH secondary to chronic kidney disease or diabetes mellitus, presence of target organ lesions, stage 2 AH with no modifiable cause and resistant AH unresponsive to lifestyle changes.

## Introduction

The causes of death and disability can be grouped into three broad categories: communicable diseases, maternal, perinatal and nutritional conditions, and non-communicable diseases. Monitoring mortality associated with these three conditions should guide health systems’ actions, promoting responses across multiple sectors and strengthening the various levels of health prevention. The result of this integrated action translates into a reduction in preventable deaths and agility in the event of changes in epidemiological circumstances^1^.

The latest World Health Organization (WHO) report, covering the period 2000–2019^1^, indicates that non-communicable diseases have become more prominent, while communicable diseases are declining. Ischemic heart disease emerged as the leading cause of death in the period 2000–2019, accounting for the largest increase in deaths - more than 2 million - in the past two decades^1^.

An estimated 17.9 million people died from cardiovascular disease (CVD) in 2019^1^, representing 32% of all deaths^1^. Of these deaths, 85% were due to ischemic heart disease and stroke. More than three-quarters of CVD deaths occur in low- and middle-income countries^1^.

Eight risk factors (alcohol use, tobacco use, high blood pressure, overweight/obesity, hypercholesterolemia, diabetes mellitus, a diet low in fruit and vegetables and high in salt, and a sedentary lifestyle) are responsible for 61% of cardiovascular deaths^2^. The combination of these factors accounts for more than three-quarters of cases of ischemic heart disease, which is the primary cause of death worldwide^2^. Additionally, over 84% of the total global burden of diseases associated with these factors occurs in low- and middle-income countries^2^. Table 1 presents the American Heart Association recommendations for optimal cardiovascular health in adults and children.

**Table 1:** American heart association recommendations for optimal cardiovascular health in adults and children

Adults (=20 years old)	For children and adolescents (<20 years old)
Smoking	Smoking
Never or quit >12 months	Never
BMI	BMI
18–25 kg/m^2^	<85th percentile
Physical activity	Physical activity
≥150 min/week moderate or =75 min/week vigorous activity or;	≥60 min/day of moderate or
≥150 min/week moderate + 2x vigorous activity	vigorous activity
Healthy diet (#)	Healthy diet
Consumption of 4-5 components	Consumption of 4-5 components
Total cholesterol	Total cholesterol
<200 mg/dL with no treatment	<170 mg/dL with no treatment
BP	BP
<120 < 80 mmHg	<90th percentile
Fasting glucose	Fasting glucose
<100 mg/dL with no treatment	<100 mg/dL with no treatment

(#) based on =4.5 cups/day of fruit and vegetables, =2 servings/week of fish, =3 servings/day of whole grains, =1080 mL/week of sugary drinks and =1,500 mg/day of sodium^3^.

The development of arterial hypertension (AH), diabetes mellitus, dyslipidemia, overweight, and obesity is the clinical translation of continuous exposure to CV risk factors^1^. Conversely, there is evidence of a reduction in the risk of CVD following smoking cessation, reduced salt intake in the diet, increased consumption of fruit and vegetables, regular physical activity, and prevention of harmful alcohol consumption^2^. Health policies should promote environments conducive to making healthy choices accessible and available in order to motivate individuals to adopt and maintain healthy behaviors^1^. In Brazil, according to WHO data in 2019, ischemic heart disease is also the leading cause of death in both sexes, as shown in Table 2^3^.

**Table 2:** Deaths from ischemic heart disease, stroke, diabetes mellitus, and kidney disease per 100,000 inhabitants, male and female, brazil, 2019^3^

Causes	Male	Female
Ischemic heart disease	90.1	67.4 (+hypertensive heart disease 13.9)
Stroke	59.4	57.5
Diabetes mellitus	26.3	30.1
Kidney diseases	19.1	16.6

Children may develop hypertension due to primary or secondary causes^4,5^. Risk factors for pediatric primary hypertension are the same as those previously mentioned for adults, including adverse perinatal events, family history of hypertension, minority race/ethnicity, inadequate sleep duration (≤8 hours/night) and quality, in addition to social determinants such as poverty and multiple adverse childhood experiences^4,5^. The association between maternal cardiovascular health during pregnancy and its effect on offspring cardiovascular health was assessed in a multinational cohort study^6^. By assessing exposure to risk factors known to be associated with CVD, it was confirmed that better maternal cardiovascular health conditions at 28 weeks of pregnancy are associated with improved offspring cardiovascular health indices at ages 10 to 14 years^6^. Data compiled from longitudinal studies mapping cardiovascular risk factors^7^ and CVD from childhood to adulthood demonstrate that the diagnosis of AH in childhood, especially with multiple measurements, is associated with the risk of AH in adulthood. Furthermore, exposure to cardiovascular risk factors in childhood is a predictor of subclinical CVD in adults^7^. The child’s body mass index, family socioeconomic status, parental risk factors, as well as genetic polymorphisms, are independent predictors of adult obesity, AH and dyslipidemia^8^. Lifestyle habits in childhood starting at the age of 9 (diet, physical activity, smoking), as well as dyslipidemia, obesity, high BP, are associated with subclinical atherosclerosis^9^, carotid intima-media thickness (C-IMT) and its progression into adulthood^9^. However, modifying risk factors by reducing BMI^8^, decreasing alcohol consumption^8^, and increasing vegetable intake from childhood^8^ to adulthood have been associated with the resolution of AH in the childhood-adulthood transition, and with the clinical reversal of adverse cardiometabolic effects in individuals who become non-obese adults^7,8,9,10,11^. A recent study confirms the association between AH in adolescence and cardiovascular events with repercussions on cardiovascular morbidity and mortality in adults. It suggests that elevated BP in children and adolescents should be clinically interpreted as early CVD^12^.

Primary AH in children is usually diagnosed as a clinical examination finding in asymptomatic children with a family history of hypertension, overweight/obesity, and normal morphofunctional characteristics of the urinary system. The diagnosis of secondary hypertension is based on information from history and clinical examination, with particular emphasis on obstructive sleep apnea, heart disease, endocrinopathies, nephropathies, and renovascular disease. In addition, there is the possibility of AH associated with medication use (decongestants, caffeine, nonsteroidal anti-inflammatory drugs, medications for neurological disorders, corticosteroids, hormonal contraceptives, tricyclic antidepressants, amphetamines) or induced by the use of illicit drugs. Secondary AH should always be considered when hypertension is diagnosed in young children with any instance of hypertensive urgency or emergency, and whenever AH accompanies signs of systemic disease, sexual development disorders, or hydroelectrolytic/acid-base imbalances^13^.

## Definition

Blood pressure (BP) in children varies depending on their age, sex and height^14^. In newborns and infants, values may vary more in the first few days of life, particularly in preterm newborns, due to the influence of birth weight and maternal conditions^14^.

Starting in 1977, with a study conducted by the “Task Force on Blood Pressure in Chidren”^15^, the BP measurement in children started to be valued. Subsequent publications in 1987, 1996 and 2004^16–18^ strengthened the methodological aspects of BP measurement in children and adolescents, as well as the design of investigation and treatment protocols for pediatric AH. Except for the first two years of life, when the oscillometric methodology shows greater technical feasibility, it is preferable to perform BP measurements using the auscultatory method, while considering the child’s sex, age and height percentile^16–18^.

In 2017, the American Academy of Pediatrics (AAP) published a new guideline^13^ with modifications to the diagnosis and management of AH. It excluded from the database previously used to develop earlier guidelines^16–18^ the BP data of overweight and obese children. This new document^13^ updates reference values by modifying the blood pressure values for the diagnosis of high BP, stages 1 and 2 AH for children between 1 and 13 years of age, and by adopting adult BP reference values, according to the American Heart Association and American College of Cardiology guidelines^19^, for children over 13 years of age. Other innovations include replacing the term “pre-hypertension” with “high BP”, simplifying recommendations for preventive assessment in routine visits, improving initial management for patients diagnosed with high BP or AH, and recommending ambulatory BP monitoring (ABPM) for the final diagnosis and management of pediatric AH. These innovations suggested by the American College of Cardiology/American Heart Association and supported by the American Academy of Pediatrics (AAP) have not yet reached international consensus. However, efforts are underway to standardize the diagnosis of AH worldwide^19,20^.

Table 3 presents the updated definition of normal BP, high BP, stages 1 and 2 of AH in children and adolescents, according to age, sex, and height percentile^13^.

**Table 3:** Updated BP definition according to age group^13^

	Children from 1 to 13 years old	Children =13 years old
Normal BP	BP <p90 for age, sex and height	BP <120/<80 mmHg
High BP	BP =p90 and <p95 for age, sex and height or BP 120/80 mmHg but <p95 (whichever is lower)	BP: 120/<80 to 129/<80 mmHg
Stage 1 hypertension	BP =p95 for age, sex and height up to <p95 +12 mmHg or BP between 130/80 and 139/89 (whichever is lower)	BP 130/80 or up to 139/89 mmHg
Stage 2 hypertension	BP + 12 mmHg for age, sex and height or BP =140/90 mmHg (whichever is lower)	BP =140/90 mmHg

BP: blood pressure; P: percentile.

## Prevalence

The prevalence of pediatric AH is approximately 3.5%. It is higher in children with obesity, chronic kidney disease and a history of prematurity^21^. Epidemiological data from the United States show an increased prevalence of AH in African-American boys and adolescents^21^, as well as high BP in 10–15% of the individuals^21,22^. In obese children aged 7 to 12, the prevalence of high BP and AH is 4.7% and 1.9%, respectively^23,24^. In China, from 1995 to 2014, there was an increase in the prevalence of overweight among children aged 7 to 17, rising from 4.3% in 1995 to 18.4% in 2014. Meanwhile, the prevalence of hypertension ranged from 4.4% to 6.4% during this period. Despite significant increases in the prevalence of overweight among Chinese children from 1995 to 2014, the prevalence of AH remained relatively stable. This suggests that other independent factors may play a role in moderating the development of AH in the pediatric population^24^.

In a study conducted in Brazil with 73,399 students aged 12 to 17, the prevalence of high BP ranged from 14.5% to 29.3% in boys aged 15–17, while the prevalence of AH was 9.6%^25^, with 17.8% of the AH prevalence attributable to obesity^25^.

The changes proposed in the 2017 guideline^13^ resulted in increased prevalence of high BP and AH, with a greater correlation between high BP and target organ damage^5,26,27^.

## BP Measurement

The indirect blood pressure measurement method was developed in 1896 by Riva-Rocci^28^, and the auscultatory method in 1905, by Korotkoff^29^. In recent years, technical advancements in BP measurement have greatly improved its accuracy. This measurement has become crucial for the diagnosis and treatment of hypertension^30^. The procedures for measuring blood pressure require careful attention, which is not always observed. This often leads to inadequate assessment of the patient’s blood pressure values and, consequently, to misdiagnosis.

In children, BP measurements may vary between visits, and even within the same visit to the doctor. Generally, BP values tend to decrease with repeated measurements^18,31^.

BP shows variations associated with physical and mental activities that, acting on respiratory, diurnal and seasonal variations, generate patterns of BP behavior with higher levels during the day and a decrease of 15% to 25% in nighttime blood pressure levels^31^. Therefore, in order to confirm AH, the diagnosis depends on multiple blood pressure measurements taken at several medical visits^16^.

The current recommendation from the Clinical Practice Guideline for Screening and Management of High Blood Pressure in Children and Adolescents^13^ considers it mandatory to measure BP annually from the age of 3. In the case of obesity, kidney disease, use of medications with an effect on BP, history of coarctation of the aorta or diabetes, it should be measured at every visit. In children under 3 years of age, this measurement should be performed in situations of increased risk of developing hypertension^13^. These include prematurity, neonates with very low birth weight, or small for gestational age, or those who required neonatal intensive care; children with diseases associated with hypertension; malformation of the urinary system, urinary tract infections or use of medications with an effect on BP^13^. It is worth noting the multiplicity of clinical situations routinely faced by pediatricians that require the systematic inclusion of blood pressure measurement in pediatric physical examinations. BP varies with age, with a progressive increase in values during childhood, reaching adult values by adolescence^8^.

The most commonly used technique is casual BP measurement in office, using auscultation with an aneroid sphygmomanometer^32^. It is known that the accuracy of these measurements will influence the diagnosis and therapeutic evaluation^31^. This assessment is subject to errors, such as poor equipment calibration, anxiety or severe crying, noisy surroundings that hinder BP auscultation, and false diagnoses^31^.

In children, BP measurement requires more time available than it does in adults, also requiring a greater variety of cuff sizes^13^. The selection of the cuff should be appropriate to the child /adolescent’s arm circumference. The width of the inflatable cuff bladder should encircle 40% of the arm circumference (measured at the midpoint between the olecranon and the acromion) and the bladder length should be 80 to 100% of this measurement^13^.

Regarding the technique used, in children over 3 years old, BP should be measured with the child seated, with the right arm at heart level^16,31^, while those under 3 years old should be assessed in the supine position^13^. Auscultation of Korotkoff phase I and the disappearance of the sounds (Korotkoff phase V) correspond to systolic blood pressure (SBP) and diastolic blood pressure (DBP), respectively. If Korotkoff phase V is heard at 0 mmHg, Korotkoff phase IV (muffling of sound) should be taken as the DBP value^13^.

The prone position can be used to measure BP in lower limbs. In this case, the cuff is placed in the calf region, covering at least 2/3 of the distance between the knee and the ankle. Due to distal pulse amplification, the SBP value at this site shows an increase of 10% to 20% compared to the measurement in the brachial artery^13^.

The 2017 pediatric AH evaluation guideline allows BP screening to be performed using oscillometric techniques, with oscillometric devices validated for the pediatric population^13^. However, these oscillometric devices can be inaccurate, especially in diastolic BP, compared to the auscultatory method^33^. Therefore, if AH is suspected with the oscillometric device, confirmatory measurements should be taken using the auscultatory method^13^. A compilation of normative values for BP in neonatal period is available for neonates aged 15 days and older, with postnatal gestational age ranging from 26 to 44 weeks^34^. The validated oscillometric devices for use in the pediatric age group^30^ can be used for non-invasive BP assessment in NB and infants until they are able to cooperate with auscultatory BP measurement. The selection of cuffs for these devices should follow the same rules used for auscultatory BP measurement^13^. The list of validated oscillometric devices for children and adolescents can be found at https://bihsoc.org/bp-monitors/ (BHS); https://www.validatebp.org (US/AMA); https://hypertension.ca/bpdevices (CANADA); https://www.stridebp.org/bp-monitors (STRIDE BP, a joint initiative of ESH, ISH and the World Hypertension League).

## BP Assessment by ABPM

Current studies have demonstrated a correlation between high BP values in childhood and adolescence and target organ damage in adulthood^35,36^.

Normative outpatient definitions for ABPM values in the pediatric population are derived from studies in the normal population. Recommendations for the use of ABPM in this population are based on expert opinions rather than evidence from well-designed studies for this purpose^37^.

ABPM is considered a mandatory procedure for confirming the diagnosis of arterial hypertension (AH) in children and adolescents^13^ presenting with office measurements at a high BP level for 1 year or more, or at a stage 1 hypertension level over 3 consecutive clinic visits^13^. Additionally, ABPM is recommended for evaluating AH and the occurrence of abnormal circadian BP patterns in children and adolescents with high-risk conditions, such as chronic kidney disease, type 1 and type 2 diabetes mellitus, pre and postoperative coarctation of the aorta, solid organ transplantation, obstructive sleep apnea syndrome, obesity, suspected masked hypertension or white coat hypertension and genetic syndromes associated with AH, such as Williams syndrome, Turner syndrome and neurofibromatosis^13^. In children with chronic kidney disease, BP should be assessed using ABPM at least annually to exclude masked AH, regardless of apparent office BP control^13^.

The equipment used for measuring ABPM could be either oscillometric or auscultatory^13^. The list of validated devices for children and adolescents can be found on the websites of British and Irish Hypertension Society (https://bihsoc.org/bp-monitors/), American Medical Association (US Blood Pressure Validated Device Listing: www.validatebp.org), Hypertension Canada (https://hypertension.ca/bpdevices) or STRIDE BP, a joint initiative of the European Society of Hypertension, International Society of Hypertension and World Hypertension League (https://www.stridebp.org/bp-monitors).

In 2017, the new guideline for the diagnosis and management of pediatric AH^13^ eased the transition from adolescence to young adulthood by establishing for adolescents ≥13 years of age the same casual BP cutoff point adopted by the adult guideline^38^.

Regarding ABPM, new American guidelines for adults have included the adoption of lower thresholds to define hypertension by ABPM in adults (mean awake BP, 130/80 mmHg, equivalent to casual BP; mean nighttime BP, 110/65 mmHg; and 24-hour mean BP, 125/75 mmHg). However, similar measures had not been taken regarding normative values for pediatric ABPM, which had been subject to controversy in the literature. In a 2014 publication^37^, Flynn et al. used the 95th percentiles of BP as a cutoff point for all pediatric ages. Meanwhile, the European Society of Hypertension (ESH), in 2016^39,40^, recommended adopting the 95th percentile of BP measured by ABPM, until they were lower than the adult cutoff points in force in Europe: awake BP, 135/85 mmHg; nighttime BP, 120/70 mmHg; and 24-hour BP, 130/80 mmHg^39,40^.

Furthermore, recent evidence has shown no additive value of pressure load in risk stratification or prediction of intermediate clinical outcomes (left ventricular hypertrophy) or progression to end-stage renal disease, favoring the elimination of pressure load measurement in pediatric ABPM classification^41,42,43,44^.

The recently published new guidelines for ABPM in children and adolescents^44^ respond to these concerns by presenting new data for classifying BP measurements through ABPM. Additionally, besides favoring the transition of care from adolescent to young adult patients, it eliminates the use of the pressure load (Table 4)^44^. When office BP and ABPM BP are both within normal range, the patient should be considered normotensive. When both are abnormal, the patient is diagnosed with ambulatory hypertension. If there is a divergence between the BP measured by the two techniques, the patient has white coat AH or masked hypertension (see Table 4).

**Table 4:** Classification for office and ABPM BP in pediatric patients^45^

	Office SBP/DBP		ABPM SBP/DBP	
	<13 y	≥13 y	<13 y	≥13 y
Normal BP	<p95	<130/80	<95th percentile or cutoff values for adolescents*	<125/75 mmHg 24-h and<130/80 mmHg awake and<110/65 mmHg nighttime
White coat AH	≥p95	≥130/80		
Masked AH	<p95	<130/80	≥95th percentile or cutoff values for adolescents*	≥125/75 mmHg 24-h or≥130/80 mmHg awake or ≥110/65 mmHg nighttime
Ambulatory AH	≥p95	≥130/80		

*Includes 24-hour, awake and nighttime BP.

## Clinical Picture and Diagnosis

The diagnosis of pediatric AH is based on the confirmation of BP values ≥95th percentile at three different visits using auscultatory methodology^13^.

In many cases, AH in children and adolescents develops asymptomatically but with significant sequelae, such as increased carotid intima-media thickness, reduced arterial distensibility, retinal arteriolar narrowing^45^ and left ventricular hypertrophy (LVH), which is present in up to 40% of cases at the time of initial diagnosis^46^, and may be a precursor to arrhythmias and heart failure in adults^44^.

The investigation into the etiology of AH begins with a thorough anamnesis and collection of information from the perinatal period up to the present moment. During investigation, it is essential to inquire history of the need for neonatal or pediatric ICU admission, umbilical vein catheterization, personal history of illness or trauma, and sleep disorders, with particular attention to sleep apnea. Also important to consider the coexistence of systemic diseases, weight loss or gain, use of medications with effects on BP, such as vasoactive drugs, immunosuppressants, and steroids, as well as the use of illicit drugs, and smoking habits^13^. Other diagnoses, such as coarctation of the aorta, central nervous system alterations, and increased intracranial pressure^13^, represent less than 10% of the etiology of AH in children^13,18^.

Physical examination should be comprehensive, including palpation of pulses in all 4 limbs, detection of abdominal murmurs through abdominal auscultation, and skin changes such as neurofibromas or acanthosis nigricans^13^. The presence of hypertensive retinopathy, enlarged heart, heart failure or neurological deficit generally correlates with the chronicity and severity of hypertension^17^. The diagnostic evaluation of hypertension in children and adolescents should be adapted to the clinical picture, family history, BP value and age at presentation^17,47^. Secondary hypertension usually occurs in younger children, with markedly elevated BP values, with 60% to 90% of cases caused by parenchymal or obstructive nephropathy or renal artery stenosis^13^.

Renovascular hypertension (RVH) is a potentially reversible cause of secondary hypertension. It may be caused by partial or total, unilateral or bilateral renal artery stenosis (RAS) or its branches, triggering and maintaining renal ischemia. Evaluation with renal Doppler ultrasound is the recommended non-invasive method for screening this clinical situation, with estimated sensitivity and specificity of 75% and 90%, respectively^48^. Magnetic resonance angiography (MRA) by digital subtraction or BOLD method or spiral CT have equal accuracy and greater sensitivity and specificity when compared to ultrasound^49^.

Endocrine disorders may account for up to 5% of AH cases, through multiple pathophysiological mechanisms, such as mineralocorticoid excess, corticoids or catecholamines; thyroid disease or hyperparathyroidism. Catecholamine-secreting tumors arising from chromaffin cells of the sympathetic-adrenal-medullary axis^50^ are characterized by the classic triad of headache, hyperhidrosis and palpitations with permanent or paroxysmal AH (50%; hypertensive peaks alternating with moments of normal BP). They could be either pheochromocytomas or paragangliomas (PPGLs), and depending on their underlying germline or somatic mutations, they could be classified into 3 groups that also differ in clinical presentation, biochemical and imaging profile^51^. Group 1 and probably group 3 tumors generally present with more aggressive symptoms and a higher metastatic risk when compared to those in group 2. Tumors from group 1 (located mainly extra-adrenal) have a noradrenergic biochemical phenotype with a tendency towards arterial hypertension, while those from group 2 (primarily located in the adrenal) have an adrenergic biochemical phenotype with intermittent secretion of catecholamines with sporadic symptoms. Plasma free metanephrine (metanephrine and normetanephrine) levels have high sensitivity (97%) and specificity (93%)^50^ for diagnosing these tumors; however, they are very expensive^50^. Urinary metanephrine measurement alone or in combination with urinary catecholamines (epinephrine, norepinephrine, and dopamine) has also been used at lower cost, although it is less sensitive. Increased values (>2 times the upper limit of normal) of urinary catecholamines indicate a high diagnostic probability^52^. Urinary catecholamines and vanillylmandelic acid levels are less sensitive for diagnosing PPGLs^53^ than urinary metanephrine levels. The location of adrenal tumors can be investigated using computed tomography, with a sensitivity of 89%, or nuclear magnetic resonance imaging (pheochromocytoma shows hyperintense signal in T2-weighted images), with a sensitivity of 98%^54^. Whole-body scintigraphy with 123I-MIBG or 68Ga DOTATE-PET-CT is highly effective in locating pheochromocytoma and paragangliomas, metastatic disease or multiple chromaffin tumors^55^. Characterizing the grouping to which the PPGL belongs, in addition to diagnostic implications, can also guide follow-up and therapeutic choices, facilitating a personalized therapeutic plan according to each patient’s specific grouping.

Monogenic AH is a cause of secondary hypertension with a familial inheritance pattern, often caused by a mutation in a single gene. It should be suspected in patients with a family history of early-onset hypertension, hypokalemia, suppressed plasma renin activity or elevated aldosterone-to-renin ratio. Among the multiple etiologies, familial hyperaldosteronism, Liddle’s syndrome and congenital adrenal hyperplasia are worth mentioning^56,57^. Genetic diagnosis may lead to appropriate treatment and enable family genetic counseling and early screening in asymptomatic family members^56^.

Primary AH is more common in older children and adolescents and is associated with overweight, obesity or a family history of AH. However, considering the wide range of secondary hypertension etiologies that may affect children or adolescents, the diagnosis of primary AH should be made with caution. In the medical history, detailed information should be provided on birth, growth and development, personal history of kidney, urological, endocrine, cardiac and neurological diseases and lifestyle habits, as well as the use of medication and other substances that may alter BP. Family history of AH, kidney disease, and other cardiovascular risk factors should also be investigated. As part of the physical examination, it is important to calculate body mass index^58^, and look for signs of secondary AH^59^. Diagnostic investigation with more invasive tests should be conducted in children under 6 years of age with signs of secondary AH^13,16^.

Sleep studies, using polysomnography, are indicated for children and adolescents with sleep disorders detected by anamnesis^13^.

Laboratory and imaging tests requested in the investigation of AH aim to define the etiology (primary or secondary), and detect target organ damage (TOD) and cardiovascular risk factors associated with AH (Chart 1)^13^. Target organ assessment should be performed in cases of stages 1 and 2 AH.

**Chart 1:** Initial investigation of children and adolescents with AH^13^

Complete blood count
Kidney function and electrolytes (including calcium, phosphorus and magnesium)
Lipid profile
Serum uric acid
Type 1 urinalysis and urine culture
Fundoscopy
Chest X-ray
Doppler echocardiography
Renal and urinary tract US with Doppler of renal arteries

## Treatment

In pediatric patients with BP values equal to or greater than the 90th percentile, non-pharmacological guidelines should be followed, focusing on weight reduction, physical exercise and dietary intervention^18,60^. This is because weight reduction in obese children and adolescents has been shown to be important in the treatment of AH and in the cardiovascular prognosis of adults^8,9^.

Regular physical activity, that is, 30 to 60 minutes of moderate physical exercise, daily if possible, has a major impact on reducing weight and blood pressure, with a better effect on systolic blood pressure than on diastolic blood pressure^61,62^. Resistance training, apart from weightlifting, can be performed by hypertensive children, but competitive sports are not recommended for patients with stage 2 hypertension^63^.

In symptomatic AH patients with secondary forms of chronic kidney disease or diabetes mellitus, presence of target organ damage, stage 2 AH and resistant AH not responsive to lifestyle changes^13^, pharmacological therapy should be initiated with an antihypertensive agent at its lowest dose, and gradually increased until BP is reduced below the 90th percentile^13^. In general, children have experienced few adverse events from antihypertensive agents^13,64^, and in the short term the use of all classes of antihypertensive agents seems to be safe^64^. International guidelines recommend as first line treatment the use of angiotensin-converting enzyme inhibitors, angiotensin receptor blockers, long-acting calcium channel blockers or thiazide diuretics. In cases of resistant AH, alpha blockers, beta blockers, centrally acting sympatholytics or potassium-sparing agents can be used^13,64^. The choice of antihypertensive medication should be made in line with the underlying pathophysiology, considering the comorbidities present in each case^65,66^. Strict dietary guidance with reduced salt intake, improved adherence to drug treatment and optimization of antihypertensive drugs should be instituted in all patients with AH, especially those with resistant AH^13^. In the event of non-response to monotherapy for longer than 6 months, referral to a specialist in pediatric AH is considered^67^.

## Conclusion

This article highlights the importance of early diagnosis of hypertension and high blood pressure in children and adolescents, emphasizing the peculiarities and difficulties of measuring blood pressure in pediatrics, as well as the importance of secondary hypertension in this age group. AH is a frequently asymptomatic condition, and it tends to progress with structural and/or functional changes in target organs such as the heart, brain, kidneys and vessels. AH is the major modifiable risk factor with an independent, linear, and continuous association for cardiovascular diseases, chronic kidney disease and premature death. The diagnosis and treatment of cardiovascular risk factors in childhood have a significant impact on reducing cardiovascular morbidity and mortality in adults.
