# Supplementary material for: Pediatric hypertension as an early manifestation of cardiovascular disease in children
Source: J Bras Nefrol. 2024 May 3;46(4):e20230159. doi: 10.1590/2175-8239-JBN-2023-0159en (PMC11197939; doi:10.1590/2175-8239-JBN-2023-0159en)
Supplement: Translation

# Translation: Hipertensão arterial pediátrica como manifestação precoce de doença cardiovascular na criança

**DOI:** 10.1590/2175-8239-JBN-2023-0159pt

**Authors:** Vera Hermina Kalika Koch, Erika Arai Furusawa

**Affiliations:** 1 Universidade de São Paulo, Faculdade de Medicina, Departamento de Pediatria, São Paulo, SP, Brasil. Universidade de São Paulo Faculdade de Medicina Departamento de Pediatria São Paulo SP Brasil; 2 Universidade de São Paulo, Faculdade de Medicina, São Paulo, SP, Brasil. Universidade de São Paulo Faculdade de Medicina São Paulo SP Brasil

**Keywords:** Hipertensão, Criança, Anormalidades Cardiovasculares

## Abstract

Em adultos, eventos cardiovasculares associados à hipertensão arterial (HA) apresentam grande repercussão na morbimortalidade. À luz dos novos conhecimentos, a HA na criança tem sido interpretada como doença cardiovascular (DCV) precoce, enquanto a exposição da criança aos fatores de risco CV revela-se preditora de DCV subclínica em adultos. As classificações da medida de pressão arterial (PA) no adulto e na faixa pediátrica foram recentemente atualizadas pelo American College of Cardiology/American Heart Association. A HA primária na criança é em geral assintomática, e se associa com história familiar de HA, sobrepeso/obesidade e normalidade morfofuncional do sistema urinário. Quanto mais jovem a criança e mais elevada a PA, maior a chance de se tratar de HA secundário. A investigação da etiologia da HA inicia-se com anamnese detalhada, que deve incluir informações clínicas e de utilização de medicamentos, fumo e álcool, desde o período perinatal até o momento da consulta. A modificação de fatores de risco, com redução do peso, redução do consumo de álcool e aumento do consumo de vegetais, entre a infância e a idade adulta, mostrou associação com a resolução da HA na transição infância-idade adulta e com a reversão dos efeitos adversos cardiometabólicos nos indivíduos adultos não obesos. A terapêutica farmacológica deve ser iniciada para casos de HA sintomática, HA secundária a doença renal crônica ou a diabetes mellitus, presença de lesões de órgão alvo, HA estágio 2 sem causa modificável e HA persistente não responsiva à mudança de estilo de vida.

## Introdução

As causas de morte e incapacidade podem ser agrupadas em três grandes categorias: enfermidades transmissíveis, condições maternas, perinatais e nutricionais, e enfermidades não transmissíveis. A monitorização da mortalidade associada a essas três condições deve nortear as ações dos sistemas de saúde promovendo respostas em múltiplos setores e fortalecendo os vários níveis de prevenção à saúde. O resultado dessa ação integrada se traduz em redução de mortes evitáveis e agilidade em casos de mudanças nas circunstâncias epidemiológicas^1^.

O último relatório da Organização Mundial de Saúde (OMS), no período 2000–2019^1^, aponta que as enfermidades não transmissíveis se tornaram mais proeminentes, enquanto as doenças transmissíveis estão em declínio. A doença isquêmica cardíaca destacou-se como a principal causa de morte no período 2000–2019, sendo responsável pelo maior aumento de mortes – mais de 2 milhões – nas últimas duas décadas^1^.

Estima-se que 17,9 milhões de pessoas morreram de doenças cardiovasculares (DCV) em 2019^1^, representando 32% de todas as mortes globais^1^. Dessas mortes, 85% foram devido a doença isquêmica cardíaca e acidente vascular cerebral. Mais de três quartos das mortes por DCV ocorrem em países de baixo e médio rendimento^1^.

Oito fatores de risco (uso de álcool, uso de tabaco, hipertensão arterial, sobrepeso/obesidade, hipercolesterolemia, diabetes mellitus, dieta pobre em frutas e vegetais e com alto conteúdo de sal, e sedentarismo) são responsáveis por 61% mortes cardiovasculares^2^. A combinação desses fatores responde por mais de três quartos dos casos de doença isquêmica cardíaca, a principal causa de morte em todo o mundo^2^, e mais de 84% da carga global total de doenças a eles associadas ocorre em países de baixa e média renda^2^. A Tabela 1 apresenta as recomendações da *American Heart Association* para saúde cardiovascular ideal de adultos e crianças.

**Tabela 1:** Recomendações da *american heart association* para saúde cardiovascular ideal de adultos e crianças

Adultos (≥20anos)	Para crianças e adolescentes (<20 anos)
Tabagismo	Tabagismo
Nunca ou abandono >12 meses	Nunca
IMC	IMC
18–25 kg/m^2^	<Percentil 85
Atividade física	Atividade física
≥150 min/semana moderado ou =75 min/semana vigorosa ou	≥60 min/dia de atividade moderada ou
≥150 min/semana moderado + 2x vigorosa	vigorosa
Dieta saudável (#)	Dieta saudável
Consumo de 4-5 componentes	Consumo de 4-5 componentes
Colesterol total	Colesterol total
<200 mg/dL sem tratamento	<170 mg/dL sem tratamento
PA	PA
<120 < 80 mmHg	<Percentil 90
Glicemia de jejum	Glicemia de jejum
<100 mg/dL sem tratamento	<100 mg/dL sem tratamento

(#) baseada em ≥4,5 xícaras/dia de frutas e vegetais, ≥2 porções/semana de peixe, ≥3 porções/dia de grãos integrais, ≤080 mL/semana de bebidas açucaradas e ≤1.500 mg/dia de sódio^3^.

O desenvolvimento de hipertensão arterial (HA), diabetes mellitus, dislipidemia, sobrepeso e obesidade constitui-se na tradução clínica à exposição contínua a fatores de risco CV^1^. Por outro lado, demonstra-se redução no risco de DCV após a cessação do consumo de tabaco, a redução do uso de sal na dieta, o aumento no consumo de frutas e vegetais, atividade física regular e a prevenção do consumo nocivo de álcool^2^. As políticas de saúde devem promover ambientes propícios para tornar escolhas saudáveis acessíveis e disponíveis para motivar as pessoas a adotarem e manterem comportamentos saudáveis^1^. No Brasil, segundo os dados da OMS em 2019, a doença cardíaca isquêmica é também a causa mais importante de óbito, em ambos os sexos, como demonstrado na Tabela 2^3^.

**Tabela 2:** Mortes por doença cardíaca isquêmica, acidente vascular cerebral, diabetes mellitus e doença renal por 100.000 habitantes, sexo masculino e feminino, brasil, 2019^3^

Causa	Homens	Mulheres
Doença cardíaca isquêmica	90,1	67.4 (+cardiopatia hipertensiva 13.9)
Acidente Vascular Cerebral	59,4	57.5
Diabetes mellitus	26,3	30.1
Doenças renais	19,1	16.6

A criança pode desenvolver hipertensão arterial por causa primária ou secundária^4,5^. Fatores de risco para hipertensão primária pediátrica são os mesmos acima citados para o adulto, acrescidos de eventos perinatais adversos, história familiar de hipertensão, raça/etnia minoritária, duração do sono ≤8 horas/noite e má qualidade do sono, além de determinantes sociais, como situação de pobreza e múltiplas experiências adversas na infância^4,5^. A associação entre a saúde cardiovascular da gestante e seu efeito na saúde cardiovascular da prole foi avaliada em estudo de coorte multinacional^6^, através da avaliação de exposição a fatores de risco reconhecidamente associados a DCV, confirmando-se que melhores condições de saúde cardiovascular materna às 28 semanas de gestação se associam a melhores índices de saúde cardiovascular da prole nas idades de 10 a 14 anos^6^. Dados compilados de estudos longitudinais que mapearam fatores de risco cardiovascular^7^ e DCV da infância à idade adulta mostram que o diagnóstico de HA na infância, principalmente com múltiplas medidas, está associado ao risco de HA no adulto, e que a exposição a fatores de risco cardiovascular na infância é preditora de DCV subclínica em adultos^7^. O índice de massa corpórea da criança, o nível socioeconômico familiar, fatores de risco parentais, bem como polimorfismos genéticos, são preditores independentes de obesidade, HA e dislipidemia em adultos^8^. O estilo de vida da infância a partir de 9 anos de idade (dieta, atividade física, tabagismo), assim como dislipidemia, obesidade, PA elevada, está associado com a aterosclerose subclínica^9^, espessura da camada íntima média carotídea (C-IMT) e sua progressão na idade adulta^9^. No entanto, a modificação de fatores de risco com redução do IMC^8^, redução do consumo de álcool^8^, elevação do consumo de vegetais entre a fase da infância^8^ e a idade adulta se associaram à resolução da HA na transição infância-idade adulta e à reversão clínica dos efeitos adversos cardiometabólicos nos indivíduos que se tornaram adultos não obesos^7–11^. Um estudo recente confirma a associação entre HA na adolescência e eventos cardiovasculares com repercussão na morbimortalidade cardiovascular em adultos e sugere que a elevação de PA na criança e no adolescente seja clinicamente interpretada como DCV precoce^12^.

A HA primária na criança é, em geral, diagnosticada como um achado de exame clínico em criança assintomática com história familiar de hipertensão, com sobrepeso/obesidade e normalidade morfofuncional do sistema urinário. O diagnóstico da hipertensão de causa secundária se baseia em informações sobre história e exame clínico, com importância para apneia obstrutiva do sono, cardiopatia, endocrinopatias, nefropatias, doença renovascular, além da possibilidade de HAS associada a uso de medicamentos (descongestionantes, cafeína, anti-inflamatórios não hormonais, medicações para distúrbios neurológicos, corticoides, contraceptivos hormonais, antidepressivos tricíclicos, anfetaminas) ou induzida por uso de drogas ilícitas. Deve-se suspeitar de HA de etiologia secundária sempre que a HA for diagnosticada na criança pequena, em todos os casos de urgência e emergência hipertensiva, assim como na HAS associada a sinais de doença sistêmica, distúrbios da diferenciação sexual ou distúrbios hidroeletrolíticos/ácido-básicos^13^.

## Definição

O valor da pressão arterial (PA) na criança sofre variação dependendo da idade, do sexo e da altura^14^. Em recém-nascidos e lactentes, esse valor pode apresentar maior variação nos primeiros dias de vida, principalmente nos recém-nascidos pré-termo devido à influência do peso de nascimento e às condições maternas^14^.

A partir de 1977, com o estudo da “Task Force on Blood Pressure in Chidren”^15^, a medida da PA em crianças começou a ser valorizada. Outras publicações se seguiram em 1987, 1996 e 2004^16–18^, fortalecendo os aspectos metodológicos da medida de PA na criança e no adolescente, assim como o delineamento da investigação e do tratamento da HA pediátrica. A exceção dos primeiros dois anos de vida, durante os quais a metodologia oscilométrica apresenta maior viabilidade técnica, a medida de PA deve ser preferencialmente realizada através do método auscultatório, levando-se em consideração sexo, idade e percentil de altura da criança^16–18^.

No ano de 2017, a Academia Americana de Pediatria (AAP) publicou nova diretriz^13^ com modificações no diagnóstico e manejo da HA, excluindo da base de dados previamente utilizada para construção das diretrizes anteriores^16–18^ os dados de PA de crianças com sobrepeso e obesidade. Esse novo documento^13^ atualiza valores de referência através de modificações nos valores pressóricos para diagnóstico de PA elevada, HA estágio 1 e 2 para crianças de 1 a 13 anos de idade e da adoção de valores referenciais de PA de adulto, segundo as diretrizes da Associação Americana de Cardiologia e do Colégio Americano de Cardiologia^19^, para as crianças maiores de 13 anos de idade. Outras inovações incluem substituição do termo pré-hipertensão por PA elevada, simplificação das recomendações na avaliação preventiva em consultas de rotina, aperfeiçoamento no manejo inicial dos pacientes com diagnóstico de PA elevada ou HA e recomendação da monitorização ambulatorial da PA (MAPA) para o diagnóstico final e manejo da HA pediátrica. Essas inovações sugeridas pelo American College of Cardiology/American Heart Association e apoiadas pela American Academy of Pediatrics (AAP) ainda não têm um consenso internacional, porém há esforços no sentido de se conseguir padronizar o diagnóstico da HA em nível mundial^19,20^.

A Tabela 3 apresenta a definição atualizada de PA normal, PA elevada, estágios 1 e 2 de HA na criança e no adolescente, de acordo com idade, sexo e percentil de altura^13^.

**Tabela 3:** Definição atualizada da PA de acordo com faixa etária^13^

	Crianças de 1 a 13 anos de idade	Crianças com idade ≥ 13 anos
PA normal	PA < p90 para idade, sexo e altura	PA <120/<80 mmHg
PA elevada	PA ≥ p90 e <p95 para idade, sexo e altura ou PA 120/80 mmHg mas <p95 o que for menor)	PA: 120/<80 a 129/<80 mmHg
Hipertensão estágio 1	PA ≥ p95 para idade, sexo e altura até <p95 +12 mmHg ou PA entre 130/80 até 139/89 (o que for menor)	PA 130/80 ou até 139/89 mmHg
Hipertensão estágio 2	PA + 12 mmHg para idade, sexo e altura ou PA ≥ 140/90 mmHg (o que for menor)	PA ≥ 140/90 mmHg

PA: pressão arterial; P: percentil.

## Prevalência

A prevalência de HA pediátrica é de aproximadamente 3,5%, sendo maior em crianças com obesidade, doença renal crônica e com antecedente de prematuridade^21^. Dados epidemiológicos americanos mostram um aumento da prevalência de HA em meninos e adolescentes afro-americanos^21^ e de PA elevada entre 10–15%^21,22^. Em crianças obesas entre 7 a 12 anos, a prevalência de PA elevada e HA é de 4,7% e 1,9%, respectivamente^23,24^. Na China, no período de 1995 a 2014, constatou-se, em crianças de 7 a 17 anos, aumento na prevalência de excesso de peso de 4,3%, em 1995, para 18,4%, em 2014, enquanto a prevalência de hipertensão oscilou na faixa de 4,4% a 6,4%. Apesar dos aumentos significativos na prevalência do excesso de peso entre as crianças chinesas entre 1995 e 2014, a prevalência da HA permaneceu relativamente estável, sugerindo que outros fatores independentes podem moderar o desenvolvimento de HA na população pediatrica^24^.

Em um estudo no Brasil, com 73.399 estudantes de 12 a 17 anos, a prevalência de PA elevada variou de 14,5% a 29,3%, em meninos entre 15–17 anos, e a de HA foi de 9,6%^25^, sendo que 17,8% da prevalência de HA podem ser atribuídas à obesidade^25^.

As mudanças propostas na diretriz de 2017^13^ resultaram em elevação da prevalência de PA elevada e HA com maior correlação da medida elevada da PA com lesões de órgãos-alvo^5,26,27^.

## Medida da PA

O método de medida de pressão arterial indireto foi desenvolvido, em 1896, por Riva-Rocci^28^, e o procedimento auscultatório, em 1905, por Korotkoff^29^. Nos últimos anos, o aprimoramento técnico da medida da PA permitiu um grande avanço na precisão dessa medida, que se tornou fundamental para o diagnóstico e o tratamento da hipertensão arterial^30^. Os procedimentos para sua realização exigem cuidados nem sempre observados, o que leva muitas vezes à avaliação inadequada dos valores pressóricos do paciente e, portanto, ao erro no diagnóstico.

Nas crianças, a medida de PA pode variar entre as consultas e até mesmo dentro de uma mesma visita ao médico. Geralmente os valores da PA tendem a diminuir com a repetição das aferições^18,31^.

A PA apresenta variações associadas às atividades física e mental que, atuando sobre as variações respiratórias, diurnas e sazonais geram padrões de comportamento da PA com níveis mais elevados durante o dia e queda de 15% a 25% nos níveis tensionais noturnos^31^. Desse modo, para a confirmação da HA, o diagnóstico depende de múltiplas medidas da pressão, realizadas em várias consultas médicas^16^.

A atual recomendação do *Clinical Practice Guideline for Screening and Management of High Blood Pressure in Children and Adolescent*^13^ considera obrigatória a medida da PA, anualmente, a partir dos 3 anos de idade ou em todas as consultas em caso de obesidade, doença renal, uso de medicamentos que tenham efeito na PA, antecedente de coarctação do arco aórtico ou diabetes. Nas crianças menores de 3 anos, essa medida deve ser realizada em situações de risco aumentado de desenvolvimento de hipertensão^13^, como prematuridade, neonatos com muito baixo peso ao nascer, ou pequenos para a idade gestacional, ou que necessitaram de cuidados intensivos neonatais; crianças portadoras de doenças que cursem com hipertensão; má-formação do sistema urinário, infecções do trato urinário ou uso de medicamentos que tenham efeito sobre a PA^13^. Note-se a multiplicidade de situações clínicas rotineiramente encontradas pelo pediatra que requerem a inclusão sistemática da medida de pressão arterial no exame físico pediátrico. A PA sofre variação com a idade e ocorre um aumento progressivo dos valores na infância, atingindo valor do adulto na época da adolescência^8^.

A técnica mais utilizada é a medida casual da PA no consultório, com técnica auscultatória com esfigmomanômetro aneroide^32^. Sabe-se que a precisão dessas medidas terá impacto no diagnóstico e na avaliação terapêutica^31^. Essa avaliação está sujeita a erros, como má calibração de equipamentos, ansiedade ou choro intenso, ambiente com muito barulho, que dificulta a ausculta da PA, e falsos diagnósticos^31^.

Nas crianças, a medida da PA demanda maior disponibilidade de tempo do que a medida em adultos e necessita de uma variedade maior de tamanhos de manguitos^13^. A escolha de manguito deve ser adequada à circunferência do braço da criança/adolescente, tendo largura da bolsa inflável do manguito correspondente a 40% da circunferência do braço (medida no ponto médio entre o olecrano e o acrômio) e comprimento da bolsa inflável do manguito equivalente a 80 a 100% dessa medida^13^.

Em relação à técnica de medida da PA, para crianças maiores de 3 anos, essa medida poderá ser feita com a criança sentada, com o braço direito apoiado na altura do coração^16,31^, enquanto as menores de 3 anos devem ser avaliadas em decúbito dorsal horizontal^13^. A ausculta da fase I de Korotkoff e o último som (fase V Korotkoff) corresponde à pressão arterial sistólica (PAS) e diastólica (PAD), respectivamente. Se a fase V de Korotkoff for ouvida a 0 mmHg, deve-se considerar a fase IV de Korotkoff (abafamento do som) como o valor da PAD^13^.

O decúbito ventral pode ser utilizado para aferição da medida da PA nos membros inferiores. Nesse caso, o manguito é colocado na região da panturrilha, cobrindo pelo menos 2/3 da distância entre o joelho e o tornozelo. Devido à amplificação do pulso distal, o valor da PAS nesse local apresenta aumento de 10% a 20% em relação à medida na artéria braquial^13^.

A diretriz para avaliação pediátrica de HA de 2017 autoriza que a triagem da PA seja realizada através de técnicas oscilométricas, com dispositivos oscilométricos validados para a faixa pediátrica^13^. Porém, esses aparelhos oscilométricos podem ser imprecisos especialmente na PA diastólica em comparação com o método auscultatório^33^, por isso, se houver suspeita de HA com aparelho oscilométrico, medidas confirmatórias devem ser realizadas por metodologia auscultatória^13^. Uma compilação de valores normativos para PA no período neonatal está disponível para neonatos a partir de 15 dias de vida e idade gestacional pós-natal entre 26 a 44 semanas^34^. Os dispositivos oscilométricos validados para uso na faixa etária pediátrica^30^ podem ser utilizados para avaliação não invasiva da PA no RN e lactentes até que sejam capazes de cooperar com a medida auscultatória da PA. A escolha de manguitos para esses dispositivos deve seguir as mesmas regras utilizadas para a mensuração da PA auscultatória^13^. A lista de aparelhos oscilométricos validados para crianças e adolescentes pode ser encontrada em https://bihsoc.org/bp-monitors/ (BHS); https://www.validatebp.org (US/AMA); https://hypertension.ca/bpdevices (CANADA); https://www.stridebp.org/bp-monitors (STRIDE BP, uma iniciativa conjunta da ESH, ISH e World Hypertension League).

## Avaliação da PA pela MAPA

Os estudos atuais têm demonstrado uma relação entre valores elevados de PA na infância e adolescência com lesões em órgãos-alvo na vida adulta^35,36^.

As definições ambulatoriais normativas para os valores da MAPA na população pediátrica são derivadas de estudos na população normal, e as recomendações para utilização de MAPA nessa população são baseadas em opiniões de especialistas, e não em evidências decorrentes de estudos bem delineados para esse fim^37^.

A MAPA é considerada um procedimento obrigatório para confirmação do diagnóstico de hipertensão arterial (HA) em crianças e adolescentes^13^, com medidas de consultório em nível de PA elevada por 1 ano ou mais, ou em nível de hipertensão estágio 1, em 3 visitas clínicas consecutivas^13^, assim como para avaliação da HA e a ocorrência de padrões circadianos anormais de PA em crianças e adolescentes com condições de alto risco, como doença renal crônica, diabetes mellitus tipos 1 e 2, pré e pós-operatório de coarctação de aorta, transplante de órgãos sólidos, síndrome da apneia obstrutiva do sono, obesidade, suspeita de hipertensão mascarada ou hipertensão do avental branco e síndromes genéticas associadas à HA, como síndrome de Williams, síndrome de Turner e a neurofibromatose^13^. Na criança com doença renal crônica, a PA deve ser avaliada pela MAPA pelo menos anualmente para descartar HAS mascarada, independentemente do aparente controle da PA de consultório^13^.

O equipamento para medida da MAPA pode ser oscilométrico ou auscultatório^13^. A lista de aparelhos validados para crianças e adolescentes pode ser encontrada nas páginas da British and Irish Hypertension Society (https://bihsoc.org/bp-monitors), da American Medical Association (US Blood Pressure Validated Device Listing: www.validatebp.org), da Hypertension Canada (https://hypertension.ca/bpdevices) ou da STRIDE BP, uma iniciativa conjunta da European Society of Hypertension, International Society of Hypertension e World Hypertension League (https://www.stridebp.org/bp-monitors).

Em 2017, a nova diretriz para diagnóstico e manejo de HA pediátrica^13^ facilitou a transição entre o adolescente e o adulto jovem, estabelecendo para adolescentes ≥13 anos de idade o mesmo ponto de corte da PA casual adotado pela diretriz do adulto^38^.

Em relação à MAPA, novas diretrizes americanas para adultos incluíram a adoção de limiares mais baixos para definir hipertensão pela MAPA em adultos (PA média de vigília, 130/80 mmHg, equivalente à PA casual; PA média do sono, 110/65 mmHg; e PA média de 24 horas, 125/75 mmHg). Medidas semelhantes, no entanto, não haviam sido tomadas em relação aos valores normativos da MAPA pediátrica que apresentavam controvérsias na literatura. Flynn e colaboradores, em publicação de 2014^37^, utilizavam os percentis 95 da PA como um ponto de corte para todas as idades pediátricas, enquanto a European Society Hypertension (ESH), em 2016^39,40^, recomendava adoção do percentil 95 de PA medida por MAPA, até que os mesmos fossem inferiores aos pontos de corte do adulto, em vigor na Europa, PA na vigília, 135/85 mmHg; PA no sono, 120/70 mmHg; e PA em 24 horas, 130/80 mmHg^39,40^.

Além disso, evidências recentes não mostravam qualquer valor aditivo de carga pressórica na estratificação de risco ou previsão de desfechos clínicos intermediários (hipertrofia de ventrículo esquerdo) ou progressão para doença renal terminal, favorecendo a eliminação da medida de carga pressórica na classificação da MAPA pediátrica^41–44^.

A nova normativa para MAPA em crianças e adolescentes^44^ publicada recentemente responde a essas inquietudes apresentando novos dados para classificação da medida de PA pela MAPA, que, além de favorecer a transição de cuidado do paciente adolescente para o adulto jovem, elimina o uso da carga pressórica (Tabela 4)^44^. Quando a PA em consultório e a PA pela MAPA forem normais, o paciente deve ser considerado normotenso. Quando ambas forem anormais, o paciente tem hipertensão ambulatorial. Se houver divergência entre a PA medida pelas duas técnicas, o paciente apresenta HA avental branco ou hipertensão mascarada (vide Tabela 4).

**Tabela 4:** Classificação para PA de consultório e pela MAPA em pacientes pediátrRicos^45^

	PAS/PAD consultório		PAS/PAD MAPA	
	<13 a	≥13 a	<13 a	≥13 a
PA normal	<p95	<130/80	<percentil 95 ou valores de corte para adolescentes*	<125/75 mmHg 24-h e<130/80 mmHg vigília e<110/65 mmHg sono
HA Avental Branco	≥p95	≥130/80		
HA Mascarada	<p95	<130/80	≥percentil 95 ou valores de corte para adolescentes*	≥125/75 mmHg 24-h ou ≥130/80 mmHg vigília ou≥110/65 mmHg sono
HA Ambulatorial	≥p95	≥130/80		

*inclui PA nas 24 h, vigília e sono.

## Quadro Clínico e Diagnóstico

O diagnóstico de HA pediátrica baseia-se na confirmação de valores de PA ≥ percentil 95 em três visitas diferentes por metodologia auscultatória^13^.

Em muitos casos, a HA na criança e no adolescente desenvolve-se de modo assintomático, mas com sequelas importantes, como aumento da espessura da camada médio-intimal da carótida, redução da distensibilidade arterial, estreitamento arteriolar na retina^45^ e hipertrofia ventricular esquerda (HVE), presente em até 40% dos casos na ocasião de seu diagnóstico inicial^46^ podendo ser um precursor de arritmias e insuficiência cardíaca em adultos^44^.

A investigação da etiologia da HA inicia-se com uma anamnese minuciosa e coleta de informações desde o período perinatal até a data atual. Deve-se indagar sobre necessidade de internação em UTI neonatal ou pediátrica, passagem de cateter umbilical, antecedentes pessoais de doença ou traumatismo, distúrbios do sono, com especial atenção à apneia do sono, coexistência de doenças sistêmicas, perda ou ganho de peso, utilização de medicamentos com efeito sobre a PA, como fármacos vasoativos, imunossupressores, esteroides, uso de drogas ilícitas e fumo^13^. Outros diagnósticos, como coarctação de aorta, alterações no sistema nervoso central e aumento da pressão intracraniana^13^, representam menos de 10% da etiologia de HA em crianças^13,18^.

O exame físico deve ser detalhado, com palpação de pulsos nos 4 membros, detecção de sopro abdominal através da ausculta abdominal, alterações de pele, como neurofibromas ou acantose nigricans^13^. A presença de retinopatia hipertensiva, cardiomegalia, insuficiência cardíaca ou déficit neurológico, em geral, correlaciona-se com a cronicidade e a gravidade da hipertensão arterial^17^. A avaliação diagnóstica da hipertensão na criança e no adolescente deve ser adequada ao quadro clínico, história familiar, valor da PA e idade de apresentação^17,47^. A hipertensão de causa secundária geralmente ocorre em crianças de menor idade, com valores muito elevados de PA, sendo que 60% a 90% dos casos são causados por nefropatias parenquimatosas ou obstrutivas ou estenose de artéria renal^13^.

A hipertensão renovascular (HARV) é uma causa potencialmente reversível de hipertensão secundária. Pode ser causada por estenose parcial ou total, uni ou bilateral da artéria renal (EAR) ou de seus ramos, com desencadeamento e manutenção de isquemia renal. A avaliação com ultrassonografia renal com Doppler renal é o método não invasivo recomendado para o rastreio dessa situação clínica, com sensibilidade e especificidade estimadas respectivamente em 75% e 90%^48^. A angiografia por RNM por subtração digital ou método BOLD ou a TC espiral têm igual acurácia e maior sensibilidade e especificidade que a ultrassonografia^49^.

Distúrbios endócrinos podem ser responsáveis por até 5% dos casos de HA, através de mecanismos fisiopatológicos múltiplos, como excesso de mineralocorticoide, corticoide ou catecolaminas; doenças da tireoide ou hiperparatireoidismo. Os tumores de células cromafins do eixo simpático-adrenomedular secretores de catecolaminas^50^ caracterizam-se pela tríade clássica de cefaleia, sudorese profusa e palpitações com HA permanente ou paroxística (50%; picos hipertensivos alternados com momentos de PA normal). Podem tratar-se de feocromocitomas ou paragangliomas (PPGLs) e, na dependência de sua linha germinativa subjacente ou mutações somáticas, podem ser classificados em 3 grupamentos que também divergem na apresentação clínica, perfil bioquímico e de imagem^51^. Os tumores do grupamento 1 e provavelmente do grupamento 3 geralmente apresentam sintomas mais agressivos com maior risco metastático quando comparados com os do grupamento 2. Os tumores do grupamento 1 (de localização principalmente extra-adrenal) apresentam fenótipo bioquímico noradrenérgico com tendência a hipertensão arterial enquanto os do grupamento 2 (localizados principalmente na adrenal) apresentam fenótipo bioquímico adrenérgico com secreção intermitente de catecolaminas com sintomas esporádicos. A dosagem de metanefrinas (metanefrina e normetanefrina) plasmáticas livres apresentam boa sensibilidade (97%) e especificidade (93%)^50^ para o diagnóstico desses tumores, porém com custo muito elevado^50^. A dosagem da metanefrina urinária isolada ou associada às catecolaminas urinárias (epinefrina, norepinefrina e dopamina) tem sido também utilizada com menor custo, embora menos sensível e os valores aumentados (> 2 vezes do limite superior da normalidade) das catecolaminas urinárias indicam alta probabilidade diagnóstica^52^. A dosagem de catecolaminas urinárias e do ácido vanilmandélico tem menor sensibilidade para o diagnóstico de PPGLs^53^ do que a dosagem de metanefrinas urinárias. A localização dos tumores adrenais pode ser investigada através de tomografia computadorizada, com sensibilidade de 89% ou de ressonância nuclear magnética (feocromocitoma apresenta hipersinal em T2), com sensibilidade de 98%^54^. A cintilografia de corpo inteiro com 123I-MIBG ou 68Ga DOTATE-PET-CT é muito eficaz em localizar feocromocitoma e paragangliomas, doença metastática ou múltiplos tumores cromafins^55^. A caracterização do grupamento a que pertence o PPGL, além das implicações diagnósticas, pode também orientar o acompanhamento e a escolha terapêutica, facilitando um plano terapêutico personalizado de acordo com o grupamento específico de cada paciente.

A HA monogênica é uma causa de hipertensão secundária de herança familiar, frequentemente causada por mutação em um gene único. Deve ser suspeitada em pacientes com história familiar de hipertensão de início precoce, hipocalemia, renina plasmática suprimida ou relação aldosterona/renina elevada. Dentre as múltiplas etiologias, ressalta-se o hiperaldosteronismo familiar, síndrome de Liddle e a hiperplasia adrenal congênita^56,57^. O diagnóstico genético pode levar ao tratamento adequado e permitir o aconselhamento genético familiar e o rastreio precoce em membros assintomáticos da família^56^.

A HA primária é mais comum em crianças maiores e adolescentes e está associada a sobrepeso, obesidade ou história familiar de HA, mas, tendo em vista a grande variedade de etiologias de hipertensão secundária que podem acometer a criança ou o adolescente, o diagnóstico de HA primária deve ser firmado com cautela. Na anamnese, devem ser detalhados dados de nascimento, crescimento e desenvolvimento, antecedentes pessoais de doenças renais, urológicas, endócrinas, cardíacas e neurológicas e hábitos de vida, bem como o uso de medicamentos e outras substâncias que possam alterar a PA, investigação de antecedentes familiares para HA, doenças renais e outros fatores de risco cardiovascular. Como parte do exame físico, deve-se calcular o índice de massa corporal^58^ e procurar indícios de HA secundária^59^. A investigação diagnóstica com exames mais invasivos deve ser feita em crianças menores de 6 anos de idade com indícios de HA secundária^13,16^.

O estudo do sono, por meio da polissonografia, está indicado para crianças e adolescentes com transtorno de sono detectado pela anamnese^13^.

Os exames laboratoriais e de imagem solicitados na investigação da HA têm como objetivo definir a etiologia (primária ou secundária) e detectar lesão de órgãos-alvo (LOA) e fatores de risco cardiovascular associado à HA (Quadro 1)^13^. A avaliação de órgãos-alvo deve ser realizada nos casos de HA estágios 1 e 2.

**Quadro 1:** investigação inicial de crRianças e adolescentes com HAha^13^

Hemograma completo
Função renal e eletrólitos (incluindo cálcio, fósforo e magnésio)
Perfil lipídico
Ácido úrico sérico
Exame de urina tipo 1 e urocultura
Fundoscopia
Radiografia de tórax
Ecodopplercardiograma
US renal e de vias urinárias com Doppler de artérias renais

## Tratamento

Aos pacientes pediátricos com valores de PA iguais ou superiores ao percentil 90, deve-se introduzir orientações não farmacológicas, como redução de peso, exercício físico e intervenção dietética^18,60^, tendo em vista que, em crianças e adolescentes obesos, a redução de peso mostrou-se importante no tratamento da HA e no prognóstico cardiovascular do adulto^8,9^.

A atividade física regular, isto é, 30 a 60 minutos de exercício físico moderado, se possível diariamente, apresenta grande impacto na redução do peso e da pressão arterial, com melhor efeito na pressão sistólica do que na diastólica^61,62^. O treinamento de resistência, com exceção de levantamento de peso, pode ser realizado pela criança hipertensa, porém esportes competitivos não são recomendados para pacientes com hipertensão arterial em estágio 2^63^.

Nos pacientes com HA sintomática, com formas secundárias de doença renal crônica ou diabetes mellitus, presença de lesão de órgão-alvo, HA estágio 2 e HA persistente não responsiva à mudança de estilo de vida^13^, a terapêutica farmacológica deve ser iniciada com um agente anti-hipertensivo em sua menor dose e aumentado gradativamente até a redução da PA abaixo do percentil 90^13^. No geral, as crianças têm apresentado poucos eventos adversos dos agentes anti-hipertensivos^13,64^, e a curto prazo a utilização de todas as classes de anti-hipertensivos parece ser segura^64^. As diretrizes internacionais recomendam como primeira linha a utilização de inibidor da enzima conversora de angiotensina, bloqueador do receptor da angiotensina, bloqueador do canal de cálcio de ação longa ou diurético tiazídicos. Em casos de HA resistente, podemos utilizar alfabloqueadores, betabloqueadores, simpatolíticos de ação central ou poupadores de potássio^13,64^. A escolha da medicação anti-hipertensiva deve ser feita em consonância com a fisiopatologia envolvida, considerando as comorbidades presentes em cada caso^65,66^. O rigor na orientação dietética com redução da ingesta de sal, melhora da adesão ao tratamento medicamentoso e otimização das drogas anti-hipertensivas deve ser instituído em todos os pacientes com HA principalmente naqueles com HA resistente^13^. Em caso de não resposta à monoterapia por mais de 6 meses, considera-se o encaminhamento ao especialista em HA na criança e no adolescente^67^.

## Conclusão

Este artigo alerta para a importância do diagnóstico precoce da hipertensão arterial e pressão arterial elevada na criança e no adolescente, ressaltando as peculiaridades e dificuldades da medida da pressão arterial em pediatria, e a importância da hipertensão arterial secundária nesta faixa etária. A HA é uma condição frequentemente assintomática, e costuma evoluir com alterações estruturais e/ou funcionais em órgãos-alvo, como coração, cérebro, rins e vasos. A HA é o principal fator de risco modificável com associação independente, linear e contínua para doenças cardiovasculares, doença renal crônica e morte prematura. O diagnóstico e tratamento dos fatores de risco cardiovascular ainda na infância apresenta um impacto significativo na redução da morbimortalidade cardiovascular no adulto.
